# Acute Torsion of Appendicular Mucocele

**DOI:** 10.1155/2017/9409281

**Published:** 2017-04-27

**Authors:** Farshid Ejtehadi, James Brooks, Hebah Hassan Ali, Vardhini Vijay

**Affiliations:** ^1^Surgical Department, The Princess Alexandra Hospital, Harlow, UK; ^2^The Michael Letcher Cellular Pathology Department, The Princess Alexandra Hospital NHS Trust, Harlow, UK

## Abstract

We present the case of an 81-year-old man with a known appendicular mucocele who presented to the emergency department with acute abdominal pain. A CT scan showed a change in orientation of the previously seen ovoid mass with surrounding fat stranding suggesting torsion. An emergency laparotomy with appendicectomy and resection of the caecal pole was performed. We discuss the findings and histopathology.

## 1. Background

Mucocele of the appendix is a term used to describe an appendix that is grossly distended by mucus. The presentation of mucoceles can vary from being an incidental finding at surgery (in 50%) or imaging to having clinical features such as abdominal pain, abdominal mass, nausea, vomiting, and acute appendicitis [1].

On histopathology, different terminologies are used to address the nature of the mucocele such as mucinous adenoma, cystadenoma, or low-grade appendiceal mucinous neoplasm.

In general, a primary neoplasm of the appendix is rare and difficult to diagnose radiologically. It is found in less than 2% of appendicectomy specimens [2] and accounts for 0.5−1% of all gastrointestinal (GI) malignancies [3–5]. Malignancy within a mucocele has an excellent prognosis with over 90% survival [4, 5].

A preoperative diagnosis of an appendicular mucocele has a very important role in its management. In the literature, authors described clinical and radiological indicators to distinguish between pathologies such as appendicitis and appendicular mucocele. These include increased luminal diameter of appendix on radiological imaging, presence of microscopic haematuria, and white blood cell count.

If a mucocele is suspected, a more extensive surgical approach may be required to reduce the risk of complications and achieve better oncological outcome [4, 5]. Pseudomyxoma peritonei can occur due to rupture or invasion of the mucocele when there is a delay in intervention. This is a result of mucus containing epithelial cells leaking into the peritoneal cavity and then proliferating and producing large quantities of mucus [6].

## 2. Case Presentation

An 81-year-old man presented acutely to our general surgical department with a 5-day history of right iliac fossa pain. The pain was gradual in onset, worsening, and left him unable to walk. He also complained of diarrhoea and loss of appetite, but no nausea, vomiting, or weight loss.

On examination, he was haemodynamically stable and had a tender, palpable mass in the right iliac fossa. His inflammatory markers were raised.

**Figure 1:**
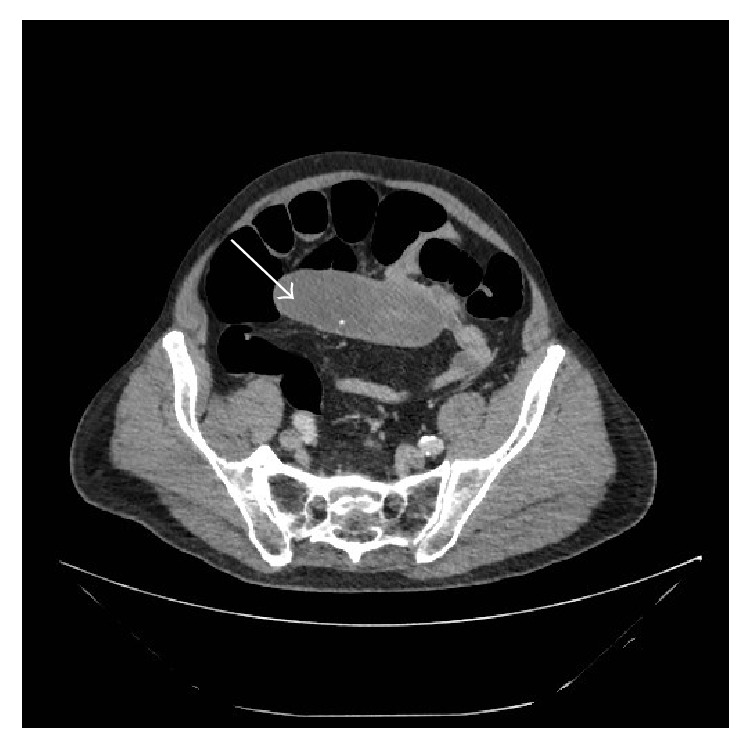
Initial CT colonogram showing a well-circumscribed ovoid soft tissue mass in the small bowel mesentery.

Few months prior to this admission, he was referred to the colorectal clinic under the 2-week referral guideline (for suspected cancer) complaining of change in his bowel habit for over 6 weeks. Considering his age, he had a CT colonogram, which had shown a well-circumscribed ovoid soft tissue mass in the small bowel mesentery at the ileocaecal junction (Figure 1). It was predominantly homogenous with soft tissue attenuation similar to muscle. There was no significant fat stranding, desmoplastic change, fibrosis, or features to suggest neoplastic change.

**Figure 2:**
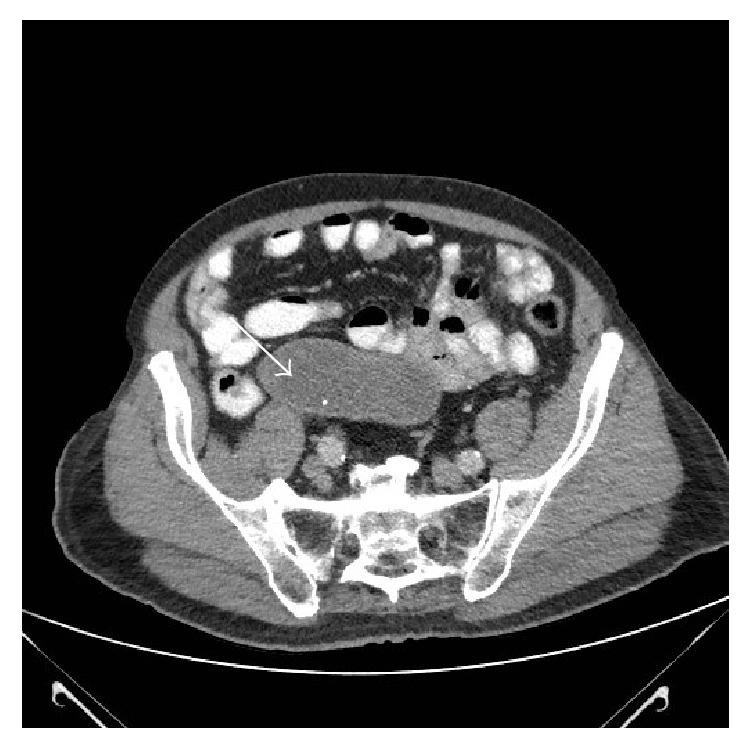
Follow-up CT abdomen pelvis showing a dilated tubular structure arising from the medial aspect of the caecum with unchanged features suggestive of a benign appendicular mucocele and no other intra-abdominal pathology.

The case was discussed in the local lower gastrointestinal multidisciplinary meeting. The patient's performance status was poor and his symptoms had settled spontaneously. As the CT did not have sinister features, no invasive surgical intervention was advised at the time. A repeat CT was recommended in 6-month time which still showed a dilated tubular structure arising from the medial aspect of the caecum with unchanged features. The suspicion of an appendicular mucocele (Figure 2) was raised. However, as the patient was still asymptomatic with a poor performance status and no neoplastic features were identified on the scan, the decision not to perform surgery was maintained.

## 3. Investigations

**Figure 3:**
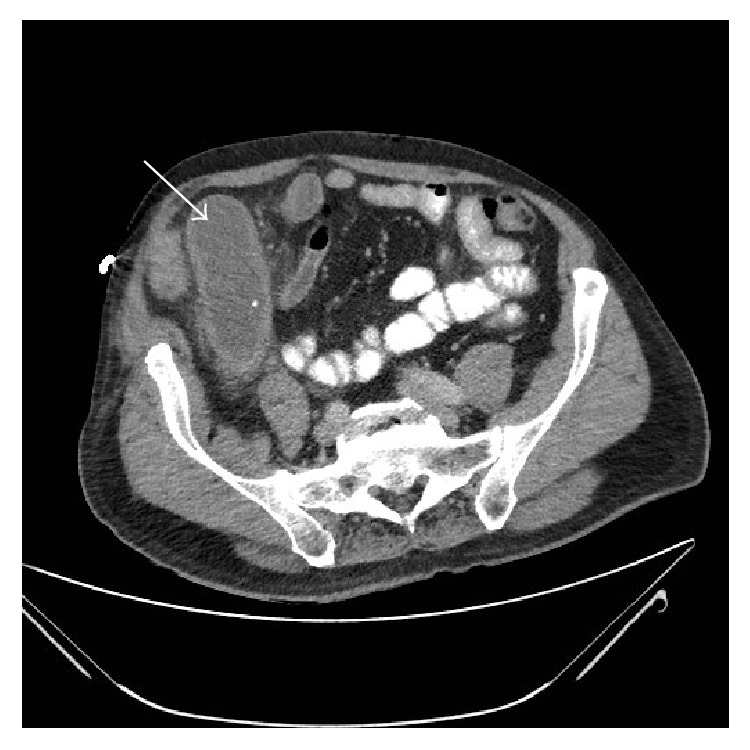
CT abdomen pelvis on admission showed a change in the morphology of the tubular cystic structure which was now vertical to transvers axis orientated with thickened walls and fat stranding suggestive of torsion or inflammation of the mucocele.

During his acute admission, however, a repeat CT scan of the abdomen showed a change in the morphology of the tubular cystic structure which was now vertical to transvers axis orientated with thickened walls and fat stranding suggestive of torsion or inflammation of the mucocele (Figure 3).

## 4. Treatment

The patient underwent a laparotomy, which revealed a large fibrotic intraperitoneal mass arising from the appendix and adherent to loops of small bowel. Adhesions to the small bowel were dissected and the mass/appendix was removed by dividing the caecal pole by a linear cutting stapler with a clear margin from the thickened appendicular base.

## 5. Outcome and Follow-Up

The patient had an uneventful postoperative recovery and was discharged home 5 days later.

**Figure 4:**
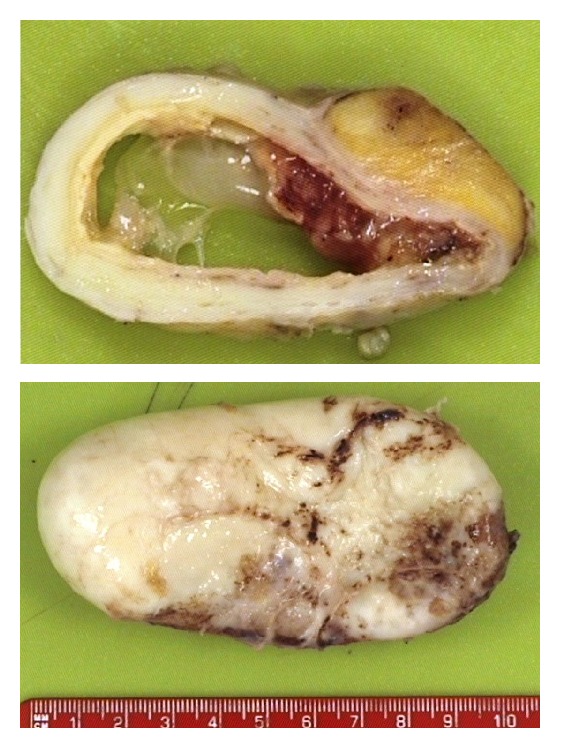
Gross appearance of the mucocele.

**Figure 5:**
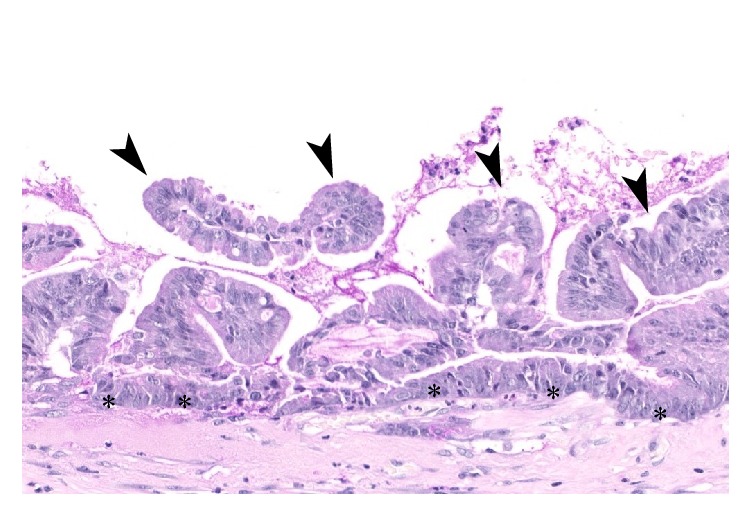
Microscopic changes: replacement of the normal appendiceal mucosa with a villiform mucinous epithelial proliferation (arrow heads): crowded columnar cells with basal with elongated, hyperchromatic nuclei (asterisks).

The specimen was sent for histopathological examination. Grossly, the appendix was cystic and dilated and had a thick wall, resembling an egg. Slicing revealed that the lumen was filled with copious viscid mucus (Figure 4). The microscopic examination (Figure 5) showed that the appendiceal mucosa was almost entirely replaced by intestinal type epithelium with low-grade dysplasia. Purulent exudate of acute inflammation was noted. The background showed chronic inflammation and fibrotic replacement of the muscularis mucosa.

Focally, displaced epithelium was identified within the fibrotic wall. However, the pathologists felt that this was likely to represent a diverticular protrusion rather than a focus of invasion as there was no desmoplastic stroma. The features were consistent with a mucinous cystadenoma.

The patient had a follow-up CT scan 3 months postoperatively which showed no evidence of recurrence or pseudomyxoma peritonei.

## 6. Discussion

Appendiceal mucinous neoplasms represent a relatively homogeneous group of neoplasms that pursue a predictable clinical course based on tumour stage and grade [7, 8]. The World Health Organization regards any neoplastic epithelial proliferation confined to the appendiceal mucosa as an adenoma. Appendiceal mucinous tumours with extra-appendiceal neoplastic epithelium are classified as mucinous adenocarcinomas and subcategorized as low or high-grade because increasingly severe cytoarchitectural atypia is associated with poorer outcome [7].

Delay in intervention may lead to rupture or invasion of the mucocele causing complications such as pseudomyxoma peritonei. 5- and 10-year survival rates are predicted at 50% and 10–30%, respectively [6].

The presentation of mucoceles can vary from being an incidental finding at surgery (in 50%) or radiology to having clinical features such as abdominal pain, abdominal mass, nausea, vomiting, and acute appendicitis [1].

Imaging findings described on plain abdominal radiographs in the literature are curvilinear right iliac fossa calcifications and mass effect on nearby structures [9]. On barium enema studies, displacement of the mesenteric side of the caecum by the appendix [10] and nonfilling of the appendix [11] are frequently found radiological features [12].

On ultrasound an appendiceal mucocele is seen as a cystic mass with a layered wall and an anechoic or hyperechoic area within the mass [13]. The “onion skin sign” first reported by Caspi et al. [14] describes a cystic mass surrounded by concentric echogenic layers and is now considered to be pathognomonic of an appendiceal mucocele.

CT appearances preoperatively usually show a round, cystic tubular mass in the location of the appendix [15]. Mucoceles are well-encapsulated with a smooth wall of variable thickness [9]. CT colonography can reveal abnormalities in the expected position of the appendiceal orifice such as an intraluminal smooth lesion or caecal contour abnormality [9].

Magnetic resonance imaging is useful for differentiating between intraperitoneal and retroperitoneal structures from large mucinous neoplasms [9]. T2 weighted pulse sequences usually show a hyperintense area within the tumour whereas T1 images are usually dependent on the mucin concentration with the signal usually being hypointense or isointense [16]. CT is more sensitive at detecting mural calcifications which is more sensitive in the diagnosis of mucinous neoplasms [16].

In an analysis of CT scans of 70 patients, the most sensitive radiological findings to suggest appendicitis with mucocele were an appendiceal lumen diameter over 13 mm and cystic dilatation and mural calcification [17]. However, differentiating between benign and neoplastic mucoceles preoperatively is more difficult. A limited study by Wang et al. in 2013 suggested that irregularity and soft tissue thickening can suggest a preoperative diagnosis of appendiceal mucocele but the study excluded patients presenting with appendicitis, ruling out a large proportion of cases [15].

Definite diagnosis is only achievable through histopathological examination of the specimen. Therefore, as the method of choice, surgical resection should be considered in cases suspected of appendicular mucocele. Depending on pathology of the mucocele, follow-up and surveillance are likely to be necessary.
